# Cortisol Acting Through the Glucocorticoid Receptor Is Not Involved in Exercise-Enhanced Growth, But Does Affect the White Skeletal Muscle Transcriptome in Zebrafish (*Danio rerio*)

**DOI:** 10.3389/fphys.2018.01889

**Published:** 2019-01-14

**Authors:** Arjan P. Palstra, Silvia Mendez, Ron P. Dirks, Marcel J. M. Schaaf

**Affiliations:** ^1^Wageningen Marine Research, Wageningen University and Research, Yerseke, Netherlands; ^2^Wageningen University & Research Animal Breeding and Genomics, Wageningen Livestock Research, Wageningen, Netherlands; ^3^Institute of Biology (IBL), Leiden University, Leiden, Netherlands; ^4^ZF-Screens BV, Leiden, Netherlands

**Keywords:** swimming exercise physiology, hypothalamic-pituitary-interrrenal (HPI) axis, cortisol, glucocorticoid receptor, RNAseq

## Abstract

Forced sustained swimming exercise at optimal speed enhances growth in many fish species, particularly through hypertrophy of the white skeletal muscle. The exact mechanism of this effect has not been resolved yet. To explore the role of cortisol, we first subjected wild-type zebrafish to an exercise protocol validated for exercise-enhanced growth, and showed that exercised zebrafish, which indeed showed enhanced growth, had higher cortisol levels than the non-exercised controls. A central role was therefore hypothesized for the steroid hormone cortisol acting through the Glucocorticoid receptor (Gr). Second, we subjected wild-type zebrafish and zebrafish with a mutant Gr to exercise at optimal, suboptimal, and super-optimal speeds and compared them with non-exercised controls. Exercised zebrafish showed growth enhancement at all speeds, with highest growth at optimal speeds. In the Gr mutant fish, exercise resulted in growth enhancement similar to wild-type zebrafish, indicating that cortisol signaling through Gr cannot be considered as a main determinant of exercise-enhanced growth. Finally, the transcriptome of white skeletal muscle tissue was analyzed by RNA sequencing. The results of this analysis showed that in the muscle tissue of Gr mutant fish a lower number of genes is regulated by exercise than in wild-type fish (183 vs. 351). A cluster of 36 genes was regulated by exercise in both wild-type and mutant fish, and in this cluster genes involved in transcriptional regulation and protein ubiquitination were overrepresented. Because these two processes appear to be regulated in both wild type and mutant fish, which both display exercise-enhanced growth, we suggest that they play an important role in the growth of muscles upon exercise.

## Introduction

Fish can be stimulated to exercise by inducing swimming behavior against a water flow. Interestingly, swimming exercise has been shown to improve the growth rate in a variety of fish species when the fish swim at speeds which can be maintained for long periods of time (Jobling et al., 1993; Davison, 1997; Palstra and Planas, 2011; Davison and Herbert, 2013). The molecular and physiological mechanisms behind this effect are still unclear. Recently, increased growth upon exercise has also been shown in the cyprinid zebrafish *Danio rerio* (Palstra et al., 2010), which has enabled research on the functional mechanisms behind exercise-enhanced growth. The advantages of this highly versatile experimental animal model will be exploited for this purpose in the present study.

This study will focus on the role of the stress hormone cortisol, since it has been shown that species such as rainbow trout (Postlethwaite and McDonald, 1995) and Atlantic salmon (Herbert et al., 2011) lower the secreted levels of cortisol under sustained exercise conditions. Cortisol is a steroid hormone that is secreted upon stress by the interrenal tissue in fish. This secretion is tightly regulated by the hypothalamic-pituitary-interrenal (HPI) axis. Upon acute stress, corticotrophin-releasing hormone (CRH) is secreted from the hypothalamus, which subsequently induces the secretion of adrenocorticotropic hormone (ACTH) from the pituitary gland, which in turn stimulates cortisol secretion from the interrenal tissue.

During stress, the effects of cortisol are mediated by an intracellular receptor, the glucocorticoid receptor (Gr). Upon activation by cortisol, the Gr acts as a transcription factor, regulating the expression of a wide variety of target genes. The zebrafish Gr is encoded by a single gene (Alsop and Vijayan, 2008; Schaaf et al., 2008) and one functional isoform is expressed (Vijayan et al., 2005; Chatzopoulou et al., 2017). Interestingly, most other fish species express two functional isoforms, Gr1 and Gr2, which are encoded by separate genes (Bury et al., 2003; Greenwood et al., 2003). The main result of the transcriptional changes induced by Gr-mediated cortisol signaling is a shift in priorities in the body by eliciting a mobilization of nutrients and oxygen to relevant organs like the heart and brain, while suppressing other systems like growth, reproduction and the immune response (Chrousos and Gold, 1992; Whirledge and Cidlowski, 2010). Considering the catabolic, growth-suppressing effects of Gr-mediated cortisol signaling on muscle tissue (reviewed by Sadoel and Vijayan, 2016), we hypothesized that the lowering of circulatory cortisol levels during exercise allows for the enhancement of muscle growth and hypertrophy.

To test this hypothesis, we have investigated in the present study whether exercise-enhanced growth originates from altered cortisol levels. First, the effects of sustained exercise on plasma cortisol levels were assessed. Second, we investigated the effect of cortisol signaling through Gr on exercise-enhanced growth at suboptimal, optimal, and super optimal swimming speeds. For this purpose, an available Gr mutant zebrafish line (Ziv et al., 2013) was used, which has a mutation in the Gr gene, effectively inhibiting the Gr-mediated effects of cortisol. Contrary to the wild-type zebrafish, Gr mutants should not experience exercise-dependent enhancement of muscle growth. Third, we have studied the transcriptional effects of cortisol during exercise-enhanced growth by RNA sequencing. Since exercise-induced growth mainly reflects hypertrophy of white skeletal muscle, we focused on this tissue. Recently we have performed transcriptome analyses (Palstra et al., 2014), which showed the activation of a series of complex transcriptional networks in white skeletal muscle of wild-type zebrafish in response to exercise. In this study, the cortisol/Gr-dependency of the exercise-induced transcriptional changes has been established.

## Materials and Methods

### Experimental Fish and Conditions

The experimental protocols complied with the current laws of the Netherlands and were approved by the animal experimental committee (DEC Nos. 2012101, 2013091, and 2013169).

Wild-type zebrafish (*D. rerio)* of 3 months old were provided by the zebrafish facilities of the Institute of Biology Leiden (Leiden, Netherlands). A Gr mutant fish line (*gr^s357^*; Ziv et al., 2013) was used which was made available by Dr. Herwig Baier (University of San Francisco). Fish of this line have a point mutation in the DNA-binding domain of the Gr gene, effectively prohibiting all Gr-mediated effects of cortisol.

Fish were housed for 2 weeks in aquaria with fresh water at 28°C at a photoperiod regime of 16L:8D and fed *ad libitum* with automatic feeders twice per day (DuplaRin pellets, Dupla, Gelsdrof, Germany). Water quality was continuously monitored and controlled.

### Experimental Set-Up and Procedure for Exercise in Zebrafish

After 2 weeks in aquaria, fish were either sampled as start group or divided into an exercise and non-exercise group (group sizes shown below). The exercise and non-exercise groups were introduced in Blazka-type 127 L swim tunnels as described by Van den Thillart et al. (2004), one tunnel for each group. The exercise group was subjected to a long-term training protocol as described by Palstra et al. (2010) which involved forced sustained exercise at swimming speeds of 0.3 m s^-1^ or ∼10 BL s^-1^ (suboptimal speed), 0.4 m s^-1^ or ∼13 BL s^-1^ (optimal swimming speed) or 0.5 m s^-1^ or ∼16 BL s^-1^ (super-optimal swimming speed), for 6 h day^-1^ (09.00–15.00 h), for 5 days week^-1^ and for 4 weeks long. The non-exercise group remained at 0.1 m s^-1^.

After three days of acclimatization in the swim tunnels, the training protocol was started. The motor speed of the tunnels with fish of the exercise group was increased slowly from resting conditions (0.1 m s^-1^) to the experimental swimming speeds. After swimming for 6 h, the speed was decreased slowly until the resting flow rate of 0.1 m s^-1^ was reached again. At the minimal flow rate of 0.1 m s^-1^ all fish could move freely in all directions, while at the flow rate of 0.4 m s^-1^ fish were forced to swim against the flow at their optimal speed. No mortality occurred among non-exercised and exercised fish due to experimental treatment.

The swim tunnels were placed in a climatized room and connected to a single storage tank holding a total fresh water volume of 500 L, recirculating continuously over a bio filter keeping water quality parameters consistent. Conditions were maintained at their photoperiod and feeding regime and at a temperature of 28°C. After the 4 weeks experimental period, exercised, and non-exercised fish were released from the tunnels. Fish were anesthetized with clove oil (dissolved 1:10 in 100% ethanol and used at a 1000 times dilution in distilled water) and measured for body weight (BW) and body length (BL; total body length TL in exp. 1, standard body length SL in exp. 2 because some fish had veil tails).

### Experimental Design

**FIGURE 1:**
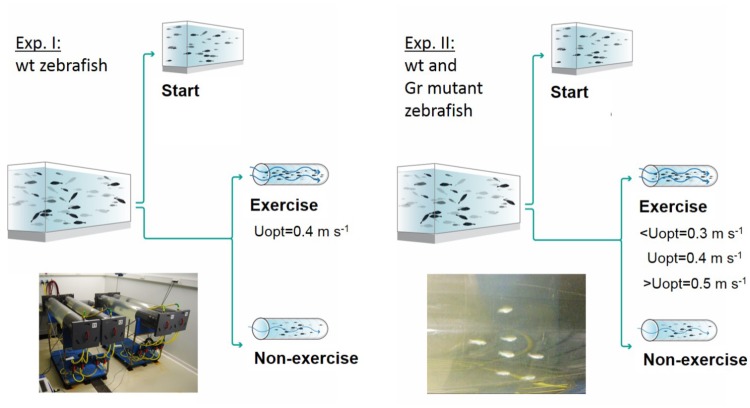
Experimental set-ups. Experiment I was executed with wild-type (wt) zebrafish with a control group sampled at the start of the experiment, a non-exercised group and an exercised group which was subjected to forced sustained swimming at the optimal swimming speed (Uopt) of 0.4 m s^-1^ for a period of 4 weeks in a swim tunnel. Experiment II had a similar set-up but was executed with wild-type zebrafish and Gr mutants. Now there were three exercise groups swimming at the sub optimal (<Uopt), optimal, and super optimal (>Uopt) swimming speeds of 0.3, 0.4, and 0.5 m s^-1^, respectively.

Two experiments were performed, and an overview of the design of these experiments is presented in Figure 1. In experiment 1, at the start of the 4 week experimental period fish were either sampled as start group (*N* = 25) or divided into an exercise and non-exercise group (each *N* = 25). The exercise group was forced to sustained exercise at an optimal swimming speed of 0.4 m s^-1^.

In experiment 2, both Gr mutant and wild-type zebrafish were either measured as start group (*N* = 10 Gr mutant and *N* = 10 wild-type fish) or divided into three exercise groups and a non-exercise group (each *N* = 15 Gr mutant and *N* = 15 wild-type zebrafish). The three exercise groups were subjected to exercise at sub-optimal swimming speed (0.3 m s^-1^ or ∼10 BL s^-1^), optimal swimming speed (0.4 m s^-1^ or ∼13 BL s^-1^) or super-optimal swimming speed (0.5 m s^-1^ or ∼16 BL s^-1^), while the non-exercise group remained at 0.1 m s^-1^.

### Cortisol Measurements Using ELISA

To determine plasma cortisol concentrations, the tails of the fish in experiment 1 were cut and blood was extracted with heparinized micro capillaries. Micro capillaries were then spun for 5 min at 11,000 rpm in a micro centrifuge. Sufficient blood could only be collected for larger fish: *N* = 5 fish of the start group; *N* = 6 non-exercised fish, and *N* = 9 exercised fish. After centrifugation, the plasma was collected in Eppendorf tubes and stored at –80°C for cortisol measurement. Cortisol plasma concentrations were determined by ELISA (Demeditec Diagnostics GmbH, Kiel-Wellsee, Germany), according to the manufacturer’s instructions.

### RNA Isolation From White Muscle Tissue

After the 4 weeks experimental period in experiment 2, the white muscle fillet was dissected dorsally from the lateral line in the epaxial quadrant along the whole length of the fish and stored in RNAlater (Ambion) at –20°C. Tissue was lysed in QIAzol Lysis Reagent. A Qiagen TissueRuptor was used to cut up the tissue samples and RNA was extracted using the Qiagen miRNeasy Mini Kit according to the manufacturer’s description (Qiagen Benelux BV, Venlo, Netherlands). RNA was eluted in 50 μl and quantified by Nanodrop (Thermo Fisher Scientific, Amsterdam, Netherlands). Integrity of the RNA was confirmed using an Agilent Bioanalyzer 2100 total RNA Nanoseries II chip (Agilent, Amstelveen, Netherlands).

### Genotyping

Fish were genotyped after experiment 2 so the SL and BW data could be assigned as belonging to Gr mutant or wild-type zebrafish. Extracted total RNA was reverse-transcribed using the iScript^TM^ cDNA Synthesis Kit (Bio-Rad). The cDNA samples were used for genotyping using a custom-designed Kompetitive Allele Specific PCR (KASP) genotyping assay. Finally, *N* = 48 Gr mutant (*N* = 13 non-exercised; *N* = 11 optimal exercised; *N* = 11 suboptimal exercised; *N* = 13 super optimal exercised) and *N* = 45 wild-type (*N* = 12 non-exercised; *N* = 11 optimal exercised; *N* = 10 suboptimal exercised; *N* = 12 super optimal exercised) zebrafish were identified.

### RNAseq: Library Preparation and Sequencing

RNAseq was performed on RNA of fish that rested (*N* = 3) or swam at Uopt (*N* = 3) for each of both Gr mutant and wild-type zebrafish groups to make all cross comparisons possible. Illumina RNAseq libraries were prepared from 1 μg total RNA using the Illumina TruSeq RNA Sample Prep Kit v2 according to the manufacturer’s instructions (Illumina, San Diego CA, United States). All RNAseq libraries (150–750 bp inserts) were sequenced on an Illumina HiSeq2500 sequencer as 1 × 50 nucleotides single-end reads according to the manufacturer’s protocol. Image analysis and base calling were done using the Illumina pipeline. Total yield varied from ∼9 to ∼16 M reads per sample.

### RNAseq: Data Analysis

Illumina reads were aligned against the zebrafish genome sequence (GRCz10.80) using TopHat (version 2.0.5; Trapnell et al., 2009). Secondary alignments of reads were excluded by filtering the files using SAMtools (version 0.1.18; Li et al., 2009). Aligned fragments per predicted gene were counted from SAM alignment files using the Python package HTSeq (version 0.5.3p9; Anders et al., 2015). In order to make comparisons across samples possible, these fragment counts were corrected for the total amount of sequencing performed for each sample. As a correction scaling factor, library size estimates determined using the R/Bioconductor (release 2.11) package DESeq (Anders and Huber, 2010) were employed. Read counts were normalized by dividing the raw counts obtained from HTSeq by its scale factor. Detailed read coverage for individual genes was extracted from the TopHat alignments using SAMtools. Raw RNAseq data (reads) have been submitted to NCBI’s Gene Expression Omnibus (GEO) as GSE120253 study with sample accession numbers GSM3396885 – GSM3396896^1^.

Comparisons were made between Uopt and non-exercised groups for both Gr mutant and wild-type zebrafish. This way, the molecular regulation of physiological processes that characterize the effects of exercise in the white skeletal muscle could be determined. Then, these exercise effects were compared between Gr mutant and wild-type zebrafish in order to identify the communalities and differences between mutants and wild-type zebrafish in response to exercise. Particularly, it is expected that this will raise insight into the role of the Gr in the response to exercise. Gene expression of differentially expressed genes, both up- or downregulated, was functionally characterized and classified using DAVID 6.8 (The Database for Annotation, Visualization and Integrated Discovery, Huang et al., 2009a,b).

### Statistics Biometric Data

Statistical analyses were carried out with IBM SPSS Statistics 22.0. Datasets were first tested for normal distribution with Kolmogorov–Smirnov tests and all data were normally distributed. As for experiment I, one-way ANOVA was performed with Tukey *post hoc* testing. As cortisol exponentially increased, log transformed data were tested. As for experiment II, treatments for each of the groups (wild-type or Gr mutant) were tested similarly. Responses to exercise were determined for each treatment vs. the controls at the start within each group, and then pairwise compared between groups with Student *t*-tests. Differences at *P* ≤ 0.05 were considered significant. Data are presented as averages ± standard errors (AV ± SE).

## Results

### Exercise-Enhanced Growth and Cortisol Levels in Wild-Type Zebrafish

**FIGURE 2:**
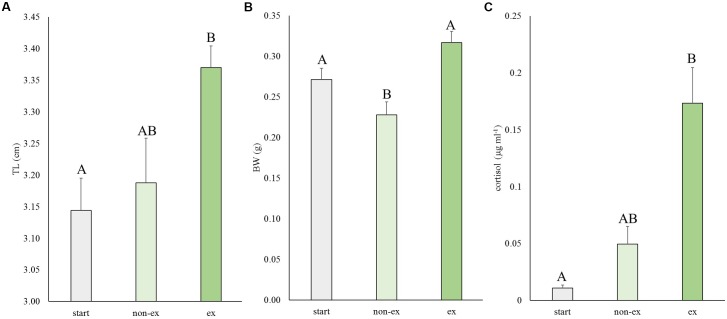
Experiment I: Exercise effects on size and cortisol levels. **(A)** Total length (TL), **(B)** body weight (BW), and **(C)** cortisol levels in wild-type fish at the start of the experiment and after 4 weeks without or with subjection to the daily exercise protocol (non-ex/ex). Differences in letters mark significant differences (*P* < 0.05).

In the first experiment, the effects of exercise at optimal swimming speed were tested on size and cortisol levels (Figure 2). Exercise effects were highly significant for length (*P* = 0.007), weight (*P* = 0.001) and cortisol levels (*P* = 0.009). Exercised fish were significantly longer than fish at the start of the experiment (*P* = 0.006; 7.3%). They were 5.6% longer than non-exercised fish but this difference was not significant (*P* = 0.062; Figure 2A). Exercised fish were significantly heavier (39.1%) than non-exercised fish: 0.32 ± 0.01 vs. 0.23 ± 0.02 g, respectively (*P* < 0.001; Figure 2B). Non-exercised fish tended to have a lower weight when compared to the fish of the start group (*P* = 0.063).

The average cortisol levels were 3.5-fold higher in exercised fish vs. non-exercised fish (0.173 ± 0.031 vs. 0.049 ± 0.016 μg ml^-1^, respectively) but due to high individual variation this difference was not significant. Cortisol concentrations of exercised fish were significantly different (*P* = 0.007) from cortisol data of fish of the start group (0.011 ± 0.003 μg ml^-1^).

### Exercise-Enhanced Growth in Wild-Type and Gr Mutant Zebrafish at Different Swimming Speeds

**FIGURE 3:**
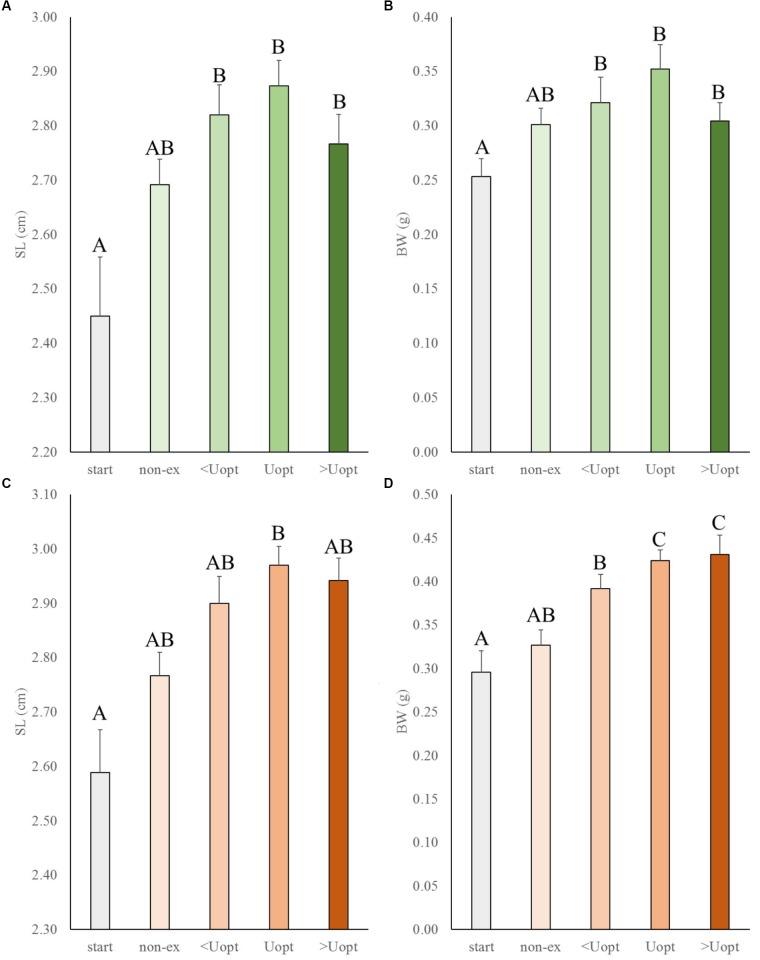
Experiment II: Exercise effects on size in wild-type and Gr mutant zebrafish. **(A)** Standard length (SL) and **(B)** body weight (BW) in wild-type zebrafish (green), and **(C)** SL and **(D)** BW of Gr mutant zebrafish (orange), at the start of the experiment (start), after 4 weeks without (non-ex) and with subjection to the daily exercise protocol swimming at the suboptimal (<Uopt), optimal, and super optimal (>Uopt) swimming speeds of 0.3, 0.4, and 0.5 m s^-1^, respectively. Differences in letters mark significant differences (*P* < 0.05).

In the second experiment, the effects of exercise at suboptimal, optimal, and super optimal swimming speed were tested on size of Gr mutant and wild-type zebrafish (Figure 3). In general, exercise enhanced growth similarly in the mutant and wild-type fish. Both groups increased in length and weight over time under exercise conditions (group and treatment effects were highly significant for length, *P* < 0.001 for both groups, and weight, *P* = 0.016 for wt and *P* < 0.001 for Gr mutants, without significant interaction). The growth patterns indicated most pronounced growth when swimming at Uopt (*P* < 0.01). Gr mutants swimming at Uopt were on average 0.12 g (*P* < 0.001; 40%) heavier than the controls at the start and 0.09 g (*P* = 0.018; 27%) heavier than the non-exercised fish. Despite the fact that Gr mutants are bigger than wt fish, comparison of treatment effects between both groups showed that groups similarly responded with growth to exercise. Only Gr mutants swimming at super optimal speed showed a significantly different response in weight than the wild type fish that were swimming at this speed (*P* = 0.008).

### Transcriptome Analysis of Muscle Tissue in Wild-Type and Gr Mutant Zebrafish During Exercise-Enhanced Growth

**FIGURE 4:**
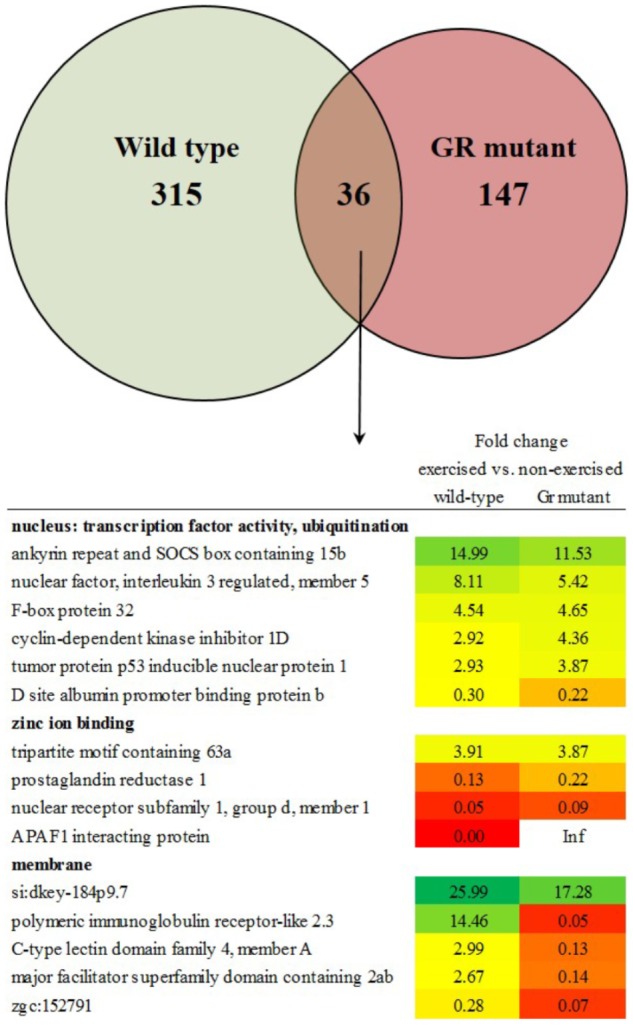
Enriched functional categories of differentially expressed genes (DEGs) specific for wild-type zebrafish, for Gr mutants and the DEGs that both groups had in common. Also shown is the functional annotation clustering of the commonly expressed genes.

The transcriptome of white skeletal muscle tissue was analyzed by using RNA sequencing. RNA was collected from wild type and Gr mutant fish, and for both groups non-exercised and exercised (Uopt) fish were used, yielding four experimental groups. In wild type fish, the comparison exercised vs. non-exercised showed significant differential expression of 351 genes at *P* < 0.05 (1.10%). Of these 351 differentially expressed genes (DEGs), expression of 192 genes was upregulated and expression of 159 genes was downregulated by exercising. In Gr mutants, this comparison showed significant differential expression of 183 genes at *P* < 0.05 (0.57%). Of these 183 DEGs, expression of 49 genes was upregulated and expression of 134 genes was downregulated upon exercise. Comparing exercised and non-exercised fish, the wild-type fish and the Gr mutants have only 36 DEGs in common (Figure 4 and Supplementary Material S1). Of these DEGs, expression of 20 genes was in the same direction and of 16 genes in opposite direction. The wild-type fish have 315 specific DEGs (Figure 4 and Supplementary Material S2) and the Gr mutants have 147 specific DEGs (Figure 4 and Supplementary Material S3). Gene ontology was analyzed for the common (Figure 4) and Gr mutant and wild-type zebrafish specific DEGs. Gene ontology for the 36 common DEGs revealed three annotation clusters with genes involved in transcription factor activity and protein ubiquitination (6 DEGs), zinc ion binding (4 DEGs) and membrane transport (5 DEGs). As these DEGs are shared between both groups that show exercise-enhanced growth, they represent important functions in white muscle physiology during hypertrophy.

Gene ontology for the 315 specific DEGs for wild-type zebrafish revealed 12 annotation clusters with enrichment scores > 1, with DEGs involved in the zona pellucida; steroid biosynthesis; oxygen transport, heme, and iron; collagen, and others. Gene ontology for the 147 specific DEGs for Gr mutant zebrafish revealed three annotation clusters with enrichment scores > 1, with DEGs involved in hydrolase activity; GTP binding; transport, transporter activity and transmembrane transport.

Thus, after comparing exercised and non-exercised fish, 351 DEGS were found in the wild-type fish and 183 DEGs in the Gr mutants. When focusing on DEGs involved in muscle growth and structure (Table 1), a group of 22 appropriate DEGs could be identified which were associated with growth factors, hormones and receptors; myosin and myoglobin, and collagen. Interesting is the expression of the *oxidative stress induced growth inhibitor 1*, downregulated in both groups. By far most of these genes were, however, differentially regulated when comparing Gr mutant and wild-type zebrafish: genes which were up or down regulated in one group were not significantly regulated in the other group. The most extreme examples are the expressions of *growth arrest-specific 7a* (fc 12.26) and *opioid growth factor receptor-like 1* (fc 9.28) which were upregulated in wild-type zebrafish, and *insulin-like growth factor binding protein 6a* (fc 40.26) and *growth regulation by estrogen in breast cancer 1* (fc 10.61) which were upregulated in Gr mutant fish. Furthermore, expression of several myosin related genes is upregulated in wild-type zebrafish but not in Gr mutant zebrafish. Also nine collagen types were upregulated in wild-type zebrafish but expression of one other type was upregulated in mutants. Finally, it is worth mentioning that the *progesterone receptor* was upregulated at fc 13.68 in Gr mutant zebrafish while expression was not significantly differential in wild-type zebrafish.

**Table 1:** Genes associated with muscle growth and structure which are differentially expressed in wild-type and/or Gr mutant zebrafish, exercised at Uopt in comparison with non-exercised zebrafish, as fold change.

	Fold change (exercised vs. non-exercised)	
	
Description	Wild-type	Gr mutant
fibroblast growth factor 13a	2.80	n.s.
oxidative stress induced growth inhibitor 1	0.21	0.16
growth arrest-specific 7a	12.26	n.s.
opioid growth factor receptor-like 1	9.28	n.s.
insulin-like growth factor binding protein 1a	3.04	n.s.
growth hormone receptor b	0.32	n.s.
insulin-like growth factor binding protein 6a	n.s.	40.26
growth regulation by estrogen in breast cancer 1	n.s.	6.05
		
myosin, heavy chain b	2.56	n.s.
myosin IIIA	4.32	n.s.
tropomyosin 1 (alpha)	2.70	n.s.
myoglobin	3.25	n.s.
		
collagen, type XI, alpha 2	4.33	n.s.
collagen, type VIII, alpha 1b	3.88	n.s.
collagen, type XI, alpha 1b	3.26	n.s.
collagen, type I, alpha 1a	3.24	n.s.
collagen, type I, alpha 2	3.19	n.s.
collagen, type I, alpha 1b	2.73	n.s.
procollagen, type V, alpha 1	2.57	n.s.
collagen, type VI, alpha 1	2.52	n.s.
collagen, type VI, alpha 2	2.45	n.s.
collagen, type X, alpha 1b	n.s.	10.61


## Discussion

In this study we have shown that exercise enhances growth and that optimal growth occurs at the optimal swimming speed. Moreover, we have shown that exercise increases cortisol levels in zebrafish. By applying swimming exercise physiology to a mutant zebrafish line with a functional knockout of the glucocorticoid receptor, we have investigated the role of cortisol signaling in exercise-enhanced growth. Our results show that cortisol signaling through the Gr receptor is not a main determinant of exercise-enhanced growth. However, RNAseq analysis revealed modulatory effects of cortisol, mediated by Gr, at the transcriptional level in white skeletal muscle tissue during exercise-enhanced growth, which apparently do not affect growth but most likely alter the physiology of the muscle tissue.

### Exercise Enhances Growth

The enhancement of growth by exercise in experiment 1 of this study was highly similar to previously published results (Palstra et al., 2010). Body weight was 39.1% higher in the exercised fish of this study vs. the non-exercised fish, which is very similar to the 41.1% as reported previously. Total length was 5.6% higher in the exercised fish of this study, which is identical to the length increase reported earlier. This validates this experimental procedure with the purpose to enhance growth by exercise and it confirms that zebrafish is a good model to study exercise-induced growth.

In the previous study and in experiment 1 of the present study, the body weight of the non-exercised fish had decreased after the 4 week experimental period. However, in experiment 2 of this study, the non-exercised fish in experiment 2 had increased in weight during the four-week exercise period. Apparently, growth conditions were better in the second experiment. A possible explanation for this difference could be that we briefly increased the flow in the tunnel after feeding in order to remove feed left overs and waste. As both exercised and non-exercised fish showed a better growth rate in the second experiment, the difference in growth between the two groups remained similar.

### Optimal Growth at the Optimal Swimming Speed

Exercised fish showed growth enhancement which was highest at the optimal swimming speed and lower at both sub and super-optimal speeds. This implicates that the optimal swimming speed for growth can be predicted by the optimal swimming speed as determined on basis of respirometry. The optimal swimming speed is the speed at which the lowest oxygen uptake per unit distance, or lowest cost of transport (COT), occurs. The lowest COT can be calculated by equaling the first derivative of the polynomial function of COT vs. speed to zero (Palstra et al., 2008). Hereby we functionally link oxygen consumption to growth performance allowing for high throughput quick scan tests for growth prediction by oxygen consumption. This association was also suggested by Davison and Herbert (2013) on basis of data for several salmonids and yellowtail kingfish. Thus, zebrafish can be added to the list of species that show optimal exercise-enhanced growth at the optimal swimming speed, and this is probably true for more cyprinid species like common carp. In addition, this finding supports the hypothesis that fish reap the benefits of the exercise at a certain optimal swimming speed without wasting energy on aggressive behavior (at speeds which are too low) or using excessive energy for swimming (at speeds which are too high) (Davison, 1997; Palstra et al., 2010).

### Exercise Increases Cortisol Levels

Exercise-enhanced growth is accompanied by higher cortisol levels in zebrafish than occurring at resting conditions. These results are in contrast with results in other fish species like rainbow trout (Postlethwaite and McDonald, 1995; Milligan et al., 2000) and Atlantic salmon (Boesgaard et al., 1993; Herbert et al., 2011), which secrete lower levels of cortisol under sustained exercise conditions than in non-exercised controls. Generally, high circulatory cortisol levels are associated with growth retardation (reviewed by Wendelaar Bonga, 1997). For example, fish subjected to stress-induced increases of cortisol levels show significantly decreased growth rates, even when food intake levels are maintained (Barnes, 2006). But this association may be context-dependent and restricted to situations of distress, while sustained exercise at optimal speeds may increase cortisol levels but still represent a eustress situation (Korte et al., 2005).

### Gr-Mediated Cortisol Signaling Does Not Affect Skeletal Muscle Growth *per se* but Alters Its Transcriptional Regulation

As exercise-enhanced growth occurred similarly in wild-type fish and Gr mutants, we conclude that Gr-mediated cortisol signaling does not affect skeletal muscle growth *per se*. Cortisol signaling through Gr can therefore not be considered as a main determinant of exercise-enhanced growth. The conclusion is also supported by the fact that salmonids show exercise-enhanced growth in combination with decreased cortisol levels (Postlethwaite and McDonald, 1995; Herbert et al., 2011) and a cyprinid like zebrafish in combination with increased cortisol levels (this study). On the other hand, it could be argued that growth enhancement in the Gr mutant is due to residual receptor activity mediating some cortisol effects, but these effects appear to be very subtle (Facchinello et al., 2017).

However, even though exercise-enhanced growth at optimal swimming speed was similar between wild-type and Gr mutant zebrafish, the effects of exercise in wild-types and mutants on muscle gene expression were highly different. The differentially regulated functional gene categories between Gr mutant and wild-type zebrafish varied greatly and reflected the functional relevance of Gr-mediated cortisol effects (e.g., Ellis et al., 2012), among others as key controller of fish intermediary metabolism and physiological homeostasis (reviews by Wendelaar Bonga, 1997; Mommsen et al., 1999) under this situation of chronic exercise stress. We therefore suggest that, although muscle growth is not affected by cortisol signaling during exercise, Gr mediated effects of this hormone have great effects on the physiology of the muscle tissue.

When comparing exercised and non-exercised fish, wild-type and Gr mutant zebrafish had 36 DEGs in common. Gene ontology analysis showed that six of these genes belonged to the cellular component *nucleus* and the functional categories protein ubiquitination and transcription factor activity. Since wild-type and Gr mutant zebrafish show similar growth upon exercise, this common category probably represents processes that are crucial for muscle growth. Protein ubiquitination is a protein degradation pathway consisting of the strongly expressed and upregulated expression of genes such as *ankyrin repeat and SOCS box containing 15b* and *F-box protein 32*. The upregulation of these catabolic genes during muscle growth may be counterintuitive, but these findings are consistent with our earlier findings in zebrafish (Palstra et al., 2014), and data indicating activation of catabolic regulators in hypertrophied muscles in humans subjected to resistance training (Leger et al., 2006). These results suggest an involvement of genes involved in protein degradation and the ubiquitin proteasome pathway or other proteolytic systems in the growth of skeletal muscles upon exercise. The proteolytic processes that these genes moderate may be important in providing the muscle with amino acids under exercise. Increased protein turnover may therefore be a prerequisite for exercise-induced hypertrophy of fast muscle fibers in adult zebrafish, as previously suggested (Palstra et al., 2014). Probably even more important for the mechanism behind exercise-enhanced growth is the common differential expression of several transcription factors such as *nuclear factor, interleukin 3 regulated, member 5*; *tumor protein p53 inducible nuclear protein 1* and *D site albumin promoter binding protein b*. These transcription factors may represent the exercise fingerprint of enhanced growth and indicate the crucial pathways that are at the base of white skeletal hypertrophy as induced by exercise. The same may well be true for *oxidative stress induced growth inhibitor 1* of which the downregulated expression will lead to stimulated growth.

Remarkably, expressions of several genes which were considered as key marker genes in a recent study on exercised zebrafish by Rovira et al. (2017) were not differentially regulated in our study. Expression of *mechanistic target of rapamycin* (*mtor*), key regulator in the PI3K/AKT/mTOR pathway toward protein biosynthesis in the muscle, was high but not differentially regulated in or between exercised Gr mutant and wild-type zebrafish. Neither were the expressions of myogenic transcription factors such as *paired box protein* *pax7* and *myocyte enhancer factor* *mef2* differentially regulated, nor the expression of any of the subunits for *AMP-activated protein kinase* (*prkaa1*, *prkaa2*, *prkab1a*, *prkab1b*, *prkab2*, *prkag1*, *prkag2a*, *prkag2b*, *prkag3a*, *prkag3b*) and *peroxisome proliferator-activated receptor gamma coactivator 1-alpha* (PGC-1α; *ppargc1a*). Therefore we cannot confirm the exercise-induced activation of the PI3K/AKT/mTOR, the mTOR-MEF2 and the AMPK-PGC1α signaling pathways toward muscle growth and proliferation, myogenesis and energy homeostasis, not in wild-type fish, nor in Gr mutants. As Rovira et al. (2017) did not report on growth of their experimental fish, and in our study we find high but no differential expression of these genes, we suggest that these pathways may play a role in muscle growth in general, but not specifically in exercise-enhanced growth.

As mentioned, when comparing exercised and non-exercised fish, wild-type and Gr mutant fish had only few DEGS in common (36) and had many specific DEGs (315 and 147, respectively). Among these specific DEGs were genes encoding growth factors, proteins involved in muscle contraction (e.g., myosins, troponin, and myoglobin) and in the extracellular matrix (e.g., collagens). Most pronounced differences when comparing genes which were differentially expressed between wild-type zebrafish and Gr mutants, were on the one hand the upregulated expressions of several genes in wild-type zebrafish such as *growth arrest-specific 7a* (*gas7a*) and *opioid growth factor receptor-like 1* (*ogfrl1*), while on the other hand the expressions of *insulin-like growth factor binding protein 6a* (*igfbp6a*) and *growth regulation by estrogen in breast cancer 1* (*greb1*) were upregulated in Gr mutants. Both *Growth arrest-specific 7a* as the *Opioid growth factor receptor-like 1* appear to have suppressive effects on growth. Expression of *growth arrest-specific 7a* occurs in cells that enter a quiescent state (Ju et al., 1998) and may have a function in neuronal development (Zhang et al., 2016). The *Opioid growth factor receptor-like 1* binds the opioid growth factor, an endogenous opioid peptide which plays an inhibitory role in cell proliferation during growth (Zagon et al., 2002). *Insulin-like growth factor binding protein 6a* is considered as a negative IGF I or II regulator and therewith functions as indirect muscle growth inhibitor (reviewed by Duan et al., 2010; Fuentes et al., 2013; Vélez et al., 2017). *Growth regulation by estrogen in breast cancer 1* (GREB1) promotes growth by estrogen-dependent proliferation of (breast cancer) cells (Camden et al., 2017). Besides estradiol, it may respond to other closely related steroid hormones and their receptors such as progesterone (Camden et al., 2017) which binds to the strongly upregulated progesterone receptor. As wild-type zebrafish and Gr mutants showed similar exercise-enhanced growth, these differentially regulated genes probably alter the physiology rather than the growth of the muscle tissue. Alternatively, they may indicate compensatory mechanisms in absence of cortisol signaling but leading to the same growth.

Other signaling pathways, such as cortisol signaling through the mineralocorticoid receptor (Vijayan et al., 2005; Prunet et al., 2006; Alsop and Vijayan, 2008) and activation of compensatory growth mechanisms, may be important for exercise-enhanced growth. For example, the GH/IGF axis and/or anabolic androgens moderating the hypertrophy of the white skeletal muscle may be involved. However, the androgen receptor was not differentially expressed in our study making a major role for anabolic androgens less plausible. We did find important differential regulation of growth factor expression which supports a major role for the GH/IGF axis in exercise-enhanced growth of zebrafish. Indeed, evidence has been provided for a pivotal role of the GH/IGF axis in exercise-enhanced growth of aquaculture fish such as coho salmon, rainbow trout, and Gilthead seabream (reviewed by Vélez et al., 2017).

## Conclusion

In conclusion, cortisol signaling cannot be considered as a main mechanism behind exercise-enhanced growth. Moreover, as Gr mutants could fulfil the long term exercise protocol, even at the highest speeds, cortisol signaling cannot even be considered as crucial in the functioning of the white muscle under exercise. However, we demonstrate that cortisol has large effects on the transcriptional response of the muscle tissue to exercise, suggesting that the physiology of the muscle is altered. Finally, we identified several Gr-independent transcriptional responses to exercise, which may be at the base of exercise-enhanced growth.

## Data Availability Statement

The raw data supporting the conclusions of this manuscript will be made available by the authors, without undue reservation, to any qualified researcher.

## Author Contributions

AP and MS conceived and designed the study. AP, SM, RD, and MS acquired the data. AP, RD, and MS analyzed and interpreted the data and drafted and reviewed the manuscript. All authors read and approved the final manuscript.

## Conflict of Interest Statement

RD was employed by company ZF-Screens BV. The remaining authors declare that the research was conducted in the absence of any commercial or financial relationships that could be construed as a potential conflict of interest.

## Footnotes

1 https://www.ncbi.nlm.nih.gov/geo/query/acc.cgi?acc=GSE120253
