# Supplementary material for: Cortisol Acting Through the Glucocorticoid Receptor Is Not Involved in Exercise-Enhanced Growth, But Does Affect the White Skeletal Muscle Transcriptome in Zebrafish (Danio rerio)
Source: Front Physiol. 2019 Jan 14;9:1889. doi: 10.3389/fphys.2018.01889 (PMC6339955; doi:10.3389/fphys.2018.01889)
Supplement: Supplementary file 1 [file Data_Sheet_1.pdf]

Cortisol Acting Through The Glucocorticoid Receptor Is Not Involved In Exercise-enhanced Growth, But Does Affect The White Skeletal Muscle Transcriptome In Zebrafish (*Danio rerio*)

Arjan.P. Palstra, Silvia Mendez, Ron P. Dirks, Marcel J.M. Schaaf

Supplementary material

Supplemental material S1: Differentially expressed genes in wild-type fish and Gr mutants as a result of optimal exercise. Specified is the expression of genes that both groups have in common, with expression in fold change depicted as heat map with highest expression in green and then from yellow to lowest expression in red (blank annotations removed).

	Fold change (exercised vs non-exercised)	
Description	wild-type	Gr mutant
si:ch73-7i4.1	Inf	7.07
si:ch73-7i4.1	419.12	6.32
si:dkey-7i4.24	188.70	0.01
vitelline membrane outer layer 1 homolog a	105.82	0.00
si:ch1073-67j19.1	78.66	0.02
si:dkey-184p9.7	25.99	17.28
si:dkey-119g10.4	14.99	0.06
ankyrin repeat and SOCS box containing 15b	14.99	11.53
polymeric immunoglobulin receptor-like 2.3	14.46	0.05
si:dkey-6n3.3	13.12	0.02
chemokine (C-C motif) ligand 34b, duplicate 8	12.20	0.10
nuclear factor, interleukin 3 regulated, member 5	8.11	5.42
ankyrin repeat and SOCS box containing 2a, tandem duplicate 1	6.91	5.58
adenosine monophosphate deaminase 3b	5.41	6.32
si:ch73-106k19.5	5.14	0.15
lectin, galactoside-binding, soluble, 9 (galectin 9)-like 1	4.56	0.06
F-box protein 32	4.54	4.65
tripartite motif containing 63a	3.91	3.87
GTP binding protein 2	3.15	4.81
C-type lectin domain family 4, member A	2.99	0.13
tumor protein p53 inducible nuclear protein 1	2.93	3.87
cyclin-dependent kinase inhibitor 1D	2.92	4.36
major facilitator superfamily domain containing 2ab	2.67	0.14
D site albumin promoter binding protein b	0.30	0.22
zgc:152791	0.28	0.07
oxidative stress induced growth inhibitor 1	0.21	0.16
chymotrypsin-like elastase family, member 1	0.16	9.82
A kinase (PRKA) anchor protein 6	0.14	4.44
prostaglandin reductase 1	0.13	0.22
period circadian clock 1a	0.12	0.14
nuclear receptor subfamily 1, group d, member 1	0.05	0.09
si:dkey-240e12.6	0.04	0.00
vitellogenin 1	0.02	0.08
coagulation factor XIII, A1 polypeptide a, tandem duplicate 1	0.01	23.32
coagulation factor XIII, A1 polypeptide	0.00	10.24
APAF1 interacting protein	0.00	Inf

Supplemental material S2: Differentially expressed genes in wild-type as a result of optimal exercise. Specified is the expression of genes specific for wild-type fish, with expression in fold change depicted as heat map with highest expression in green and then from yellow to lowest expression in red (blank annotations removed).

Description	Fold change wild-type
zgc:172290	Inf
5S ribosomal RNA	Inf
si:dkey-266f7.10	Inf
chemokine (C-C motif) ligand 34a, duplicate 3	Inf
si:dkey-29d5.1	Inf
phosphate cytidylyltransferase 1, choline, beta b	Inf
protein phosphatase 1, regulatory subunit 3G	Inf
core 1 synthase, glycoprotein-N-acetylgalactosamine 3-beta-galactosyltransferase 1	150.22
RAR-related orphan receptor C b	48.39
Uncharacterized protein	34.63
U1 spliceosomal RNA	33.19
U1 spliceosomal RNA	27.54
U2 spliceosomal RNA	27.39
U2 spliceosomal RNA	24.85
nuclear factor, interleukin 3 regulated, member 2	24.51
major histocompatibility complex class I UBA	24.24
Small nucleolar RNA U3	23.10
Metazoan signal recognition particle RNA	22.56
U2 spliceosomal RNA	20.47
si:dkey-266f7.5	20.29
5.8S ribosomal RNA	18.76
forkhead box J1b	17.97
MAX dimerization protein 3	15.58
U1 spliceosomal RNA	14.50
si:dkey-119g10.3	12.51
si:ch211-5k11.6	12.33
growth arrest-specific 7a	12.26
fibronectin type III domain containing 4a	12.00
si:ch211-250g4.3	11.64
RNase MRP	11.06
solute carrier family 25 (mitochondrial folate carrier), member 32a	10.66
5.8S ribosomal RNA	10.57
si:ch211-66o10.5	10.25
methyltransferase like 11B	9.84
opioid growth factor receptor-like 1	9.28
ADAM metallopeptidase domain 8a	9.28
Nuclear RNase P	8.79
si:ch211-269m17.1	8.73
zgc:92880	8.70
neurofilament, medium polypeptide a	8.70
si:dkey-119g10.5	8.63
aryl hydrocarbon receptor nuclear translocator-like 1a	8.59
bone gamma-carboxyglutamate (gla) protein, like	6.97
5.8S ribosomal RNA	6.52
ST3 beta-galactoside alpha-2,3-sialyltransferase 2	6.47
Metazoan signal recognition particle RNA	6.13
transmembrane protein 229B	5.94

integrin, alpha M (complement component 3 receptor 3 subunit)	5.83
Metazoan signal recognition particle RNA	5.81
si:ch211-250k18.8	5.76
N-ethylmaleimide-sensitive factor attachment protein, gamma b	5.62
leucine rich repeat and fibronectin type III domain containing 5b	5.61
carbonic anhydrase	5.47
Metazoan signal recognition particle RNA	5.46
solute carrier family 35, member E4	5.45
chemokine (C-C motif) ligand 34a, duplicate 4	5.35
alkB, alkylation repair homolog 3 (E. coli)	5.31
serine/threonine/tyrosine kinase 1	5.21
carboxylesterase 3	4.98
MICAL-like 1	4.86
Metazoan signal recognition particle RNA	4.84
nuclear protein 1	4.83
kelch-like family member 34	4.80
ADP-ribosylation factor-like 2 binding protein	4.80
kelch-like family member 24b	4.72
cysteine-serine-rich nuclear protein 1a	4.67
hemoglobin, alpha adult 1	4.60
poly (ADP-ribose) polymerase family, member 12a	4.55
yippee-like 3	4.55
Cbp/p300-interacting transactivator, with Glu/Asp-rich carboxy-terminal domain, 2	4.52
si:xx-by187g17.1	4.38
collagen, type XI, alpha 2	4.33
myosin IIIA	4.32
nuclear protein, transcriptional regulator, 1-like	4.30
transferrin receptor 1a	4.10
solute carrier family 6 (neurotransmitter transporter, glycine), member 9	4.08
carboxylesterase 3	4.08
ba1 globin, like	4.06
secreted phosphoprotein 1	4.06
zinc finger protein 385B	4.03
ba1 globin	4.01
splA/ryanodine receptor domain and SOCS box containing 3b	4.00
aminolevulinate, delta-, synthase 2	3.97
unc-51 like autophagy activating kinase 1a	3.94
collagen, type VIII, alpha 1b	3.88
FERM domain containing 6	3.86
retinoic acid induced 14	3.85
si:dkey-263j23.1	3.84
si:ch1073-95o12.1	3.79
Uncharacterized protein	3.75
neutrophil cytosolic factor 1	3.74
si:dkey-43b14.8	3.69
erythrocyte membrane protein band 4.1b (elliptocytosis 1, RH-linked)	3.65
neuralized E3 ubiquitin protein ligase 1Ab	3.62
zgc:158463	3.57
EH-domain containing 3	3.55
si:dkeyp-3b12.6	3.55
si:dkey-77p23.6	3.53
si:dkeyp-3b12.5	3.52
pantothenate kinase 1a	3.45
si:dkeyp-3b12.7	3.45

xin actin-binding repeat containing 1	3.43
zgc:162183	3.39
ubiquitin B	3.33
peptidase domain containing associated with muscle regeneration 1	3.33
matrix-remodelling associated 8a	3.30
collagen, type XI, alpha 1b	3.26
myoglobin	3.25
collagen, type I, alpha 1a	3.24
si:ch211-246m6.4	3.22
collagen, type I, alpha 2	3.19
chromosome 19 open reading frame 12	3.17
si:dkeyp-3b12.9	3.17
headcase homolog (Drosophila)	3.16
trans-2,3-enoyl-CoA reductase a	3.15
tRNA methyltransferase 1 homolog (S. cerevisiae)-like	3.14
si:dkeyp-3b12.11	3.11
RAR-related orphan receptor A, paralog a	3.11
coiled-coil domain containing 141	3.08
zgc:92113	3.08
coagulation factor X	3.07
C-type lectin domain family 3, member B	3.06
insulin-like growth factor binding protein 1a	3.04
si:dkeyp-3b12.10	3.02
chromosome 2 open reading frame 40	3.01
zgc:136971	3.00
dual-specificity tyrosine-(Y)-phosphorylation regulated kinase 3	2.99
yippee-like 1 (Drosophila)	2.99
thioredoxin-like 4B	2.98
leucine-rich repeat LGI family, member 3	2.97
abhydrolase domain containing 3	2.97
OTU deubiquitinase 4	2.94
periostin, osteoblast specific factor b	2.93
OTU deubiquitinase 4	2.93
si:dkey-153m14.1	2.92
microtubule associated tumor suppressor 1a	2.90
si:dkeyp-3b12.8	2.82
leucine rich repeat containing 30a	2.82
metallothionein 2	2.82
fibroblast growth factor 13a	2.80
potassium voltage-gated channel, shaker-related subfamily, member 7	2.77
OTU deubiquitinase 4	2.77
si:ch211-202a12.4	2.76
OTU deubiquitinase 4	2.74
collagen, type I, alpha 1b	2.73
tropomyosin 1 (alpha)	2.70
cryptochrome 2a	2.67
pleckstrin and Sec7 domain containing 3, like	2.67
splA/ryanodine receptor domain and SOCS box containing 3a	2.66
claudin 5b	2.65
si:dkey-32n7.4	2.61
si:dkeyp-1h4.9	2.59
fibronectin type III domain containing 1	2.58
osteoglycin	2.57
procollagen, type V, alpha 1	2.57

myeloid/lymphoid or mixed-lineage leukemia (trithorax homolog, Drosophila); translocated to, 11	2.56
myosin, heavy chain b	2.56
RNA, 7SK small nuclear	2.56
collagen, type VI, alpha 1	2.52
glutamic-oxaloacetic transaminase 1, soluble	2.49
collagen, type VI, alpha 2	2.45
si:ch1073-398f15.1	0.42
karyopherin (importin) beta 3	0.40
leiomodin 3 (fetal)	0.40
methylsterol monooxygenase 1	0.40
L-threonine dehydrogenase	0.39
wu:fb13g09	0.39
ring finger and CCCH-type domains 2	0.38
DEAD (Asp-Glu-Ala-Asp) box helicase 21	0.38
adenosine monophosphate deaminase 1 (isoform M)	0.38
BCL2-related ovarian killer a	0.37
solute carrier family 22, member 14	0.36
pellino E3 ubiquitin protein ligase family member 3	0.36
myelocytomatosis oncogene homolog	0.36
zinc finger and BTB domain containing 16b	0.36
HECT and RLD domain containing E3 ubiquitin protein ligase 2	0.36
guanine nucleotide binding protein-like 2 (nucleolar)	0.34
cytokine inducible SH2-containing protein	0.34
choline kinase beta	0.33
NSA2 ribosome biogenesis homolog (S. cerevisiae)	0.33
transmembrane protein 100	0.33
keratin 97	0.33
sodium channel, voltage-gated, type IV, beta b	0.33
phosphodiesterase 4C, cAMP-specific a	0.32
growth hormone receptor b	0.32
smoothelin-like 1	0.32
thyrotrophic embryonic factor a	0.32
DEAD (Asp-Glu-Ala-Asp) box helicase 24	0.31
polyribonucleotide nucleotidyltransferase 1	0.31
asteroid homolog 1 (Drosophila)	0.31
cystathionine-beta-synthase b	0.31
pleckstrin homology-like domain, family A, member 3	0.31
zinc finger protein 395b	0.31
polymerase (DNA-directed), delta 3, accessory subunit	0.30
7-dehydrocholesterol reductase	0.30
keratin 96	0.29
aldo-keto reductase family 1, member B1 (aldose reductase)	0.29
CLOCK-interacting pacemaker b	0.29
nei endonuclease VIII-like 1 (E. coli)	0.29
glutathione peroxidase 1b	0.29
hormonally up-regulated Neu-associated kinase	0.28
si:ch1073-416d2.4	0.28
L-threonine dehydrogenase 2	0.28
circadian associated repressor of transcription b	0.28
ring finger protein 145a	0.27
hepatic leukemia factor a	0.27
3-hydroxy-3-methylglutaryl-CoA reductase a	0.26
cytochrome P450, family 51	0.25
transmembrane 7 superfamily member 2	0.25

tripartite motif containing 54	0.25
squalene epoxidase a	0.24
MID1 interacting protein 1, like	0.24
family with sequence similarity 102, member B, a	0.24
heat shock factor 2	0.24
acetylserotonin O-methyltransferase 2	0.23
DEAD/H (Asp-Glu-Ala-Asp/His) box helicase 11	0.23
solute carrier family 7 (cationic amino acid transporter, y+ system), member 1	0.23
eph receptor B6	0.23
lanosterol synthase (2,3-oxidosqualene-lanosterol cyclase)	0.23
tyrosinase-related protein 1b	0.22
24-dehydrocholesterol reductase	0.21
period circadian clock 1b	0.21
synaptonemal complex protein 2-like	0.21
methionine adenosyltransferase II, alpha b	0.20
calcium binding and coiled-coil domain 1b	0.19
cryptochrome 5	0.19
si:ch211-155m12.1	0.18
cryptochrome 3	0.18
ring finger protein 207a	0.18
adenosylmethionine decarboxylase 1	0.17
NAD(P) dependent steroid dehydrogenase-like	0.17
solute carrier family 25 (mitochondrial carnitine/acylcarnitine carrier), member 29	0.17
synaptosomal-associated protein 23.2	0.16
emopamil binding protein (sterol isomerase)	0.16
fatty acid binding protein 1b, liver, tandem duplicate 2	0.16
glutathione S-transferase kappa 1	0.15
secretory calcium-binding phosphoprotein 7	0.15
saccharopine dehydrogenase (putative)	0.15
heat shock cognate 70-kd protein, tandem duplicate 2	0.14
si:ch211-101l18.8	0.14
circadian associated repressor of transcription a	0.13
si:dkey-18a10.3	0.13
transmembrane protein 233	0.13
si:dkey-11e23.3	0.13
D site albumin promoter binding protein a	0.12
si:dkey-112e17.1	0.12
heat shock cognate 70-kd protein, tandem duplicate 1	0.12
heat shock cognate 70-kd protein, tandem duplicate 3	0.10
si:dkey-22i16.3	0.10
si:dkey-104n9.1	0.10
si:ch211-132b12.7	0.10
si:ch73-160p18.3	0.09
heat shock cognate 70-kd protein, like	0.09
cytochrome b5 reductase 2	0.08
F-box and WD repeat domain containing 10	0.08
si:ch211-12h2.6	0.07
si:dkeyp-50f7.2	0.07
vomeronasal 2 receptor, a17	0.07
zgc:111868	0.06
pyruvate kinase, liver and RBC	0.05
wu:fi42e03	0.05
cytochrome P450, family 27, subfamily C, polypeptide 1	0.05
cytochrome P450, family 7, subfamily A, polypeptide 1a	0.04

retinol saturase (all-trans-retinol 13,14-reductase) like	0.04
zona pellucida glycoprotein 3a, tandem duplicate 2	0.04
si:dkey-19b23.11	0.04
alpha-2-macroglobulin-like 1	0.04
alpha-2-macroglobulin-like 1	0.04
zona pellucida glycoprotein 3a, tandem duplicate 1	0.03
linker histone H1M	0.03
si:ch211-263m18.4	0.03
Uncharacterized protein	0.02
zgc:171779	0.02
Uncharacterized protein	0.02
si:zfos-1505d6.3	0.02
zona pellucida glycoprotein 3, tandem duplicate 2	0.01
zgc:152652	0.01
zgc:171670	0.01
zona pellucida glycoprotein 2, tandem duplicate 3	0.01
zona pellucida glycoprotein 2, tandem duplicate 1	0.01
zgc:165539	0.00
si:ch211-14a17.7	0.00
zgc:173856	0.00
vitellogenin 2	0.00
cytochrome P450, family 4, subfamily V, polypeptide 7	0.00
zgc:173770	0.00
zgc:173837	0.00
zgc:175135	0.00
zgc:171750	0.00
zgc:171781	0.00
zona pellucida glycoprotein 2, tandem duplicate 5	0.00
si:dkey-241l7.3	0.00
si:ch211-154a22.8	0.00
si:dkey-241l7.5	0.00
si:ch1073-263o8.2	0.00
si:dkeyp-46h3.2	0.00
zgc:171977	0.00
zgc:171474	0.00
si:dkey-229d11.3	0.00
Uncharacterized protein	0.00

Supplemental material S3: Differentially expressed genes in Gr mutant fish as a result of optimal exercise. Specified is the expression of genes specific for Gr mutants, with expression in fold change depicted as heat map with highest expression in green and then from yellow to lowest expression in red (blank annotations removed).

Description	Fold change Gr mutant
sc:d217	Inf
insulin-like growth factor binding protein 6a	40.26
zgc:171497	39.29
elastase 2	35.53
keratin 78, type II	29.95
chymotrypsinogen B1	26.22
carboxyl ester lipase, tandem duplicate 1	25.58
protease, serine, 59, tandem duplicate 1	23.70
trypsin	17.49
keratin 78, type II	17.48
si:ch211-240l19.8	17.41
protease, serine, 59, tandem duplicate 2	16.44
carboxyl ester lipase, tandem duplicate 2	14.52
elastase 2 like	13.59
solute carrier family 16, member 12b	12.52
si:ch211-190k17.19	12.30
si:ch211-89o9.4	11.89
RAR-related orphan receptor C b	11.07
amylase, alpha 2A (pancreatic)	10.93
collagen, type X, alpha 1b	10.61
elastase 3 like	9.99
methyltransferase like 21C	8.19
uncharacterized LOC100329294 (LOC100329294), mRNA	7.41
growth regulation by estrogen in breast cancer 1	6.05
si:ch73-304f21.2	5.90
kelch-like family member 38b	4.81
si:ch211-260e23.9	4.36
sphingomyelin phosphodiesterase, acid-like 3B	4.13
pyruvate dehydrogenase kinase, isozyme 2a	3.62
nuclear factor, erythroid 2-like 1a	3.58
F-box protein 40, tandem duplicate 2	3.08
6-phosphofructo-2-kinase/fructose-2,6-biphosphatase 3	2.95
si:dkey-8k3.2	0.29
chemokine (C-C motif) ligand 34b, duplicate 4	0.27
zgc:158446	0.25
serum/glucocorticoid regulated kinase 2b	0.24
si:dkey-79f11.5	0.23
mannose receptor, C type 1b	0.22
deoxyribonuclease II, lysosomal	0.22
Fc receptor, IgE, high affinity I, gamma polypeptide like	0.21
cathepsin Sb, tandem duplicate 1	0.20
si:dkeyp-9d4.2	0.20
wu:fj29h11	0.20
heat shock protein, alpha-crystallin-related, b6	0.19
membrane-spanning 4-domains, subfamily A, member 17A.9	0.19
midnolin	0.18
Uncharacterized protein	0.18

si:ch211-198c19.3	0.18
si:dkey-238c7.13	0.18
nicotinamide riboside kinase 2	0.18
ATPase, Na+/K+ transporting, alpha 1a polypeptide, tandem duplicate 4	0.18
eukaryotic translation initiation factor 4A1A	0.18
zgc:123068	0.17
granulin 1	0.17
zgc:92326	0.17
zgc:65811	0.17
Uncharacterized protein	0.17
heat shock protein, alpha-crystallin-related, 9	0.16
si:ch211-165i18.2	0.16
si:dkey-79f11.8	0.16
si:ch211-217k17.11	0.16
si:dkey-211g8.8	0.15
si:ch211-165b19.4	0.15
si:ch211-183d5.2	0.15
Uncharacterized protein	0.15
si:ch73-106k19.4	0.15
interleukin 4 induced 1	0.15
si:dkey-188i13.9	0.14
ISG15 ubiquitin-like modifier	0.14
purine nucleoside phosphorylase 5a	0.13
lectin, galactoside-binding, soluble, 2b	0.13
si:ch211-217k17.12	0.13
three-finger protein 5 (LOC100003647), mRNA	0.13
signal transducer and activator of transcription 1b	0.12
transferrin-a	0.12
major histocompatibility complex class II DAB gene	0.12
URGCP-MRPS24 readthrough	0.12
proteasome (prosome, macropain) subunit, alpha type, 6, like	0.12
aldehyde dehydrogenase 8 family, member A1	0.12
hepatitis A virus cellular receptor 1	0.11
si:dkey-79f11.10	0.11
eukaryotic translation initiation factor 4E family member 1c	0.11
PDZK1 interacting protein 1	0.10
solute carrier family 43 (amino acid system L transporter), member 2b	0.10
si:dkey-79f11.9	0.09
guanylate binding protein 1	0.09
myxovirus (influenza virus) resistance C	0.09
ankyrin repeat domain 1a (cardiac muscle)	0.09
Uncharacterized protein	0.09
fatty acid amide hydrolase	0.09
si:dkey-102g19.3	0.08
solute carrier family 5 (sodium/glucose cotransporter), member 2	0.08
phenylalanine hydroxylase	0.07
solute carrier family 5 (sodium/glucose cotransporter), member 1	0.07
thioredoxin	0.07
cyclin-dependent kinase 5, regulatory subunit 2b (p39)	0.07
myxovirus (influenza) resistance A	0.07
si:dkey-58f10.11	0.06
si:ch211-24o10.6	0.06
granzyme A	0.06
solute carrier family 6 (neutral amino acid transporter), member 19b	0.06

transmembrane protein 27	0.06
alanyl (membrane) aminopeptidase b	0.05
solute carrier family 12 (sodium/chloride transporter), member 3	0.05
lectin, galactoside-binding, soluble, 3 binding protein b	0.04
nephrosin	0.04
zgc:171731	0.04
lectin, galactoside-binding, soluble, 3 binding protein a	0.04
solute carrier family 22 (organic anion transporter), member 6, like	0.03
si:rp71-40n14.7	0.03
uncoupling protein 1	0.03
myeloid-specific peroxidase	0.03
PDZ domain containing 1	0.03
si:dkeyp-75b4.10	0.03
cadherin 17, LI cadherin (liver-intestine)	0.02
single-minded family bHLH transcription factor 1b	0.02
guanylate cyclase 2C	0.02
solute carrier family 5 (sodium/monocarboxylate cotransporter), member 12	0.02
cubilin (intrinsic factor-cobalamin receptor)	0.02
si:ch211-198c1.7	0.02
leukocyte cell-derived chemotaxin 2 like	0.02
si:ch1073-429i10.1	0.02
parvalbumin 9	0.02
lysozyme	0.01
phospholipase A2, group IVC (cytosolic, calcium-independent)	0.01
glutathione S-transferase kappa 1	0.00
steroidogenic acute regulatory protein	0.00
HNF1 homeobox Ba	0.00
formimidoyltransferase cyclodeaminase	0.00
immune-related, lectin-like receptor 4	0.00
major facilitator superfamily domain containing 4b	0.00
cytochrome P450, family 2, subfamily k, polypeptide 31	0.00
matrix metallopeptidase 13a	0.00
alkaline phosphatase, liver/bone/kidney	0.00
HNF1 homeobox Bb	0.00
solute carrier family 34 (type II sodium/phosphate cotransporter), member 1a	0.00
fatty acid binding protein 7, brain, b	0.00
cholesterol 25-hydroxylase like 2	0.00
HORMA domain containing 1	0.00
solute carrier family 22 (organic anion transporter), member 7b, tandem duplicate 1	0.00
urocanate hydratase 1	0.00
solute carrier family 22 (organic anion transporter), member 7b, tandem duplicate 2	0.00
solute carrier family 6 (neutral amino acid transporter), member 19	0.00
si:ch211-139a5.9	0.00
guanylate cyclase activator 2B (uroguanylin)	0.00
